# Advances in research on the role of gut microbiota in the pathogenesis and precision management of gallstone disease

**DOI:** 10.3389/fmed.2025.1535355

**Published:** 2025-06-25

**Authors:** Ludong Tan, Feng Jia, Yahui Liu

**Affiliations:** Department of Hepatobiliary and Pancreatic Surgery, General Surgery Center, The First Hospital of Jilin University, Changchun, China

**Keywords:** gallstone, gut microbiota, cholecystectomy, bile acid metabolism, microbiome-based therapies

## Abstract

Gallstone disease remains a prevalent global gastrointestinal condition with a rising incidence, posing substantial challenges to healthcare systems and public health initiatives. Advances in multi-omics and sequencing technologies have illuminated the pivotal role of gut microbiota in its pathogenesis, progression, and management. This article reviews the disrupted microbiota profiles observed in patients with gallstone disease and their connection to the metabolic pathways involved in gallstone formation, particularly focusing on cholesterol metabolism, bile acid dynamics, and inflammatory pathways. It also discusses the enduring impacts of cholecystectomy on gut microbial functions and their metabolic implications. Novel strategies targeting gut microbiota, including probiotics, microbial metabolite supplementation, dietary adjustments, integrative medicine, and emerging microbial therapies, present promising avenues for precise treatment. Furthermore, this review underscores the value of future research into multi-omics integration, microbiome engineering, and global collaborations, advocating for interdisciplinary and personalized approaches to gallstone management. However, unresolved challenges, such as ensuring stable colonization of functional microbiota, refining tailored therapeutic strategies, and assessing long-term outcomes, warrant further investigation. This work aims to provide a comprehensive resource for understanding gallstone disease within a microecological framework, supporting the development of precision medicine-based prevention and treatment paradigms.

## 1 Introduction

Gallstone disease, a prevalent gastrointestinal condition, poses a growing global health challenge due to its increasing incidence and substantial burden on healthcare systems. This condition, defined by the formation of cholesterol or pigment stones in the gallbladder or bile ducts, affects approximately 10–15% of adults worldwide, with higher rates in specific populations (1). Annual incidence ranges from 0.60 to 1.39% in Europe and 12–15% in North America, while in China, a prevalence of 11% significantly impacts public health (2, 3).

The pathogenesis of gallstone disease is multifactorial, involving genetic predisposition, metabolic abnormalities, lifestyle factors, and dietary patterns. The Westernization of dietary habits, characterized by high-fat and high-calorie intake, significantly elevates the risk of cholesterol metabolism disorders, thereby promoting gallstone formation (4). Although many patients remain asymptomatic, approximately 30% develop complications such as acute or chronic cholecystitis, cholangitis, or pancreatitis, leading to increased healthcare costs and reduced quality of life (3).

In recent years, advancements in high-throughput sequencing technologies (e.g., 16S rRNA sequencing, metagenomics) and multi-omics approaches (e.g., metabolomics, proteomics) have unveiled the pivotal role of the gut microbiota in the pathogenesis and progression of gallstone disease (5, 6). As early as the early 20th century, studies observed potential links between gallstones and bacterial infections, such as *Helicobacter pylori* (7). Subsequent research has revealed that specific gut microbiota, such as Desulfovibrionales, may play a critical role in gallstone formation by disrupting bile acid metabolism homeostasis (8).

The gut microbiota represents a highly complex and dynamic ecosystem in the human body, comprising thousands of microbial species. These microorganisms, including bacteria, archaea, fungi, and viruses, coexist in a delicate balance within the gastrointestinal tract. They play crucial roles in various aspects of human health and disease, such as aiding in digestion, producing essential vitamins, modulating the immune system, and influencing metabolic processes. The composition and function of the gut microbiota can be influenced by a multitude of factors, including diet, lifestyle, genetics, and environmental exposures, making it a fascinating and intricate area of study in modern medicine and biology. It is not only associated with metabolic disorders and immune regulation but also directly influences gallstone formation through its interaction with bile acid metabolism (9). For instance, the gut microbiota’s ability to metabolize bile acids significantly alters the composition and size of the bile acid pool, thereby impacting cholesterol solubility and deposition risks (10).

Despite widespread attention to the role of the gut microbiota in gallstone disease, its underlying mechanisms require further investigation. This review aims to systematically summarize recent advances in understanding the role of the gut microbiota in the onset, progression, and intervention of gallstone disease, including its structural characteristics, metabolic mechanisms, the impact of cholecystectomy, and microbiota-targeted prevention and treatment strategies, providing insights for future basic and clinical research.

## 2 Structural characteristics of the gut microbiota

The gut microbiota, one of the most complex microbial ecosystems in the human body, has attracted considerable attention from researchers due to its composition and functions. In the healthy human gut, there are over 1,000 microbial species, with a total population exceeding 100 trillion. Among these microorganisms, bacteria have the most prominent impact on body metabolism. These microorganisms are predominantly composed of Firmicutes (approximately 80%) and Bacteroidetes (approximately 10%), along with Actinobacteria and Proteobacteria (11).

Recent studies have identified significant dysbiosis in the gut microbiota of gallstone disease patients. At the phylum level, the gut microbiota in these patients remains dominated by Firmicutes, followed by Bacteroidetes, Actinobacteria, and Proteobacteria (6, 12). Table 1 summarizes research findings on the mechanisms by which gut microbiota influence gallstone formation.

**Table 1:** Studies of the gut microbiome in gallstone disease.

Ref.	Methods	Disease vs control	Samples	Microbial changes
Hu et al	16S RNA sequencing	Gallstone vs. gallstone free	Feces	↑Desulfovibrionales (8)
Song et al	16S DNA sequencing	Gallstone vs. gallstone free	Feces	↓Klebsiella, Roseburia, Collinsella, Enterobacter, Dialister; ↑Streptococcus, Lactobacillus, Romboutsia, Fusobacterium, Megamonas (13)
Wu et al	16S rRNA sequencing	Gallstone vs. gallstone free	Feces	↓Faecalibacterium, Lachnospira, Roseburia; ↑Proteobacteria (9)
Wang et al	16S rRNA sequencing	Gallstone vs. gallstone free	Feces	↓Firmicutes, the Firmicutes/Bacteroidetes (F/B) ratio; ↑Cyanobacteria, Fusobacteria, Spirochaetes (6)
Wells et al.	Culture	Gallstone vs. gallstone free	Feces	↑7alpha-Dehydroxylating bacteria (16)
Keren et al	16S rRNA sequencing	Gallstone vs. gallstone free	Feces	↓Roseburia; ↑Oscillospira (14)

Using 16S rRNA sequencing, Wang et al. compared the gut microbiota of 30 gallstone disease patients with 30 healthy controls and found a significant decrease in the proportion of Firmicutes and the Firmicutes/Bacteroidetes (F/B) ratio in patients. At the genus level, the abundance of probiotics, including Lactobacillus and Bifidobacterium, was significantly reduced (6). Song et al. used 16S DNA sequencing to compare the gut microbiota of gallstone patients and healthy individuals. Their study revealed that asymptomatic gallstone patients had significantly lower levels of Klebsiella, Roseburia, Collinsella, Dialister, and Enterobacter, while Streptococcus, Lactobacillus, Romboutsia, Fusobacterium, and Megamonas were increased (13). Wu et al. were the first to use 16S rRNA sequencing to compare the gut microbiota of 29 gallstone patients and 38 healthy controls. Their results showed a significant increase in Proteobacteria levels in gallstone patients, along with a marked reduction in Lachnospira, Faecalibacterium, and Roseburia (9).

Emerging research have focused on the functional genetic alterations in the gut microbiota of patients with gallstone disease, particularly genes linked to bile acid metabolism. Notably, the expression levels of critical enzyme genes, such as bile salt hydrolase (BSH) and bile acid dehydroxylase, are significantly altered, potentially disrupting bile acid balance and contributing to gallstone formation (14).

**Figure 1:**
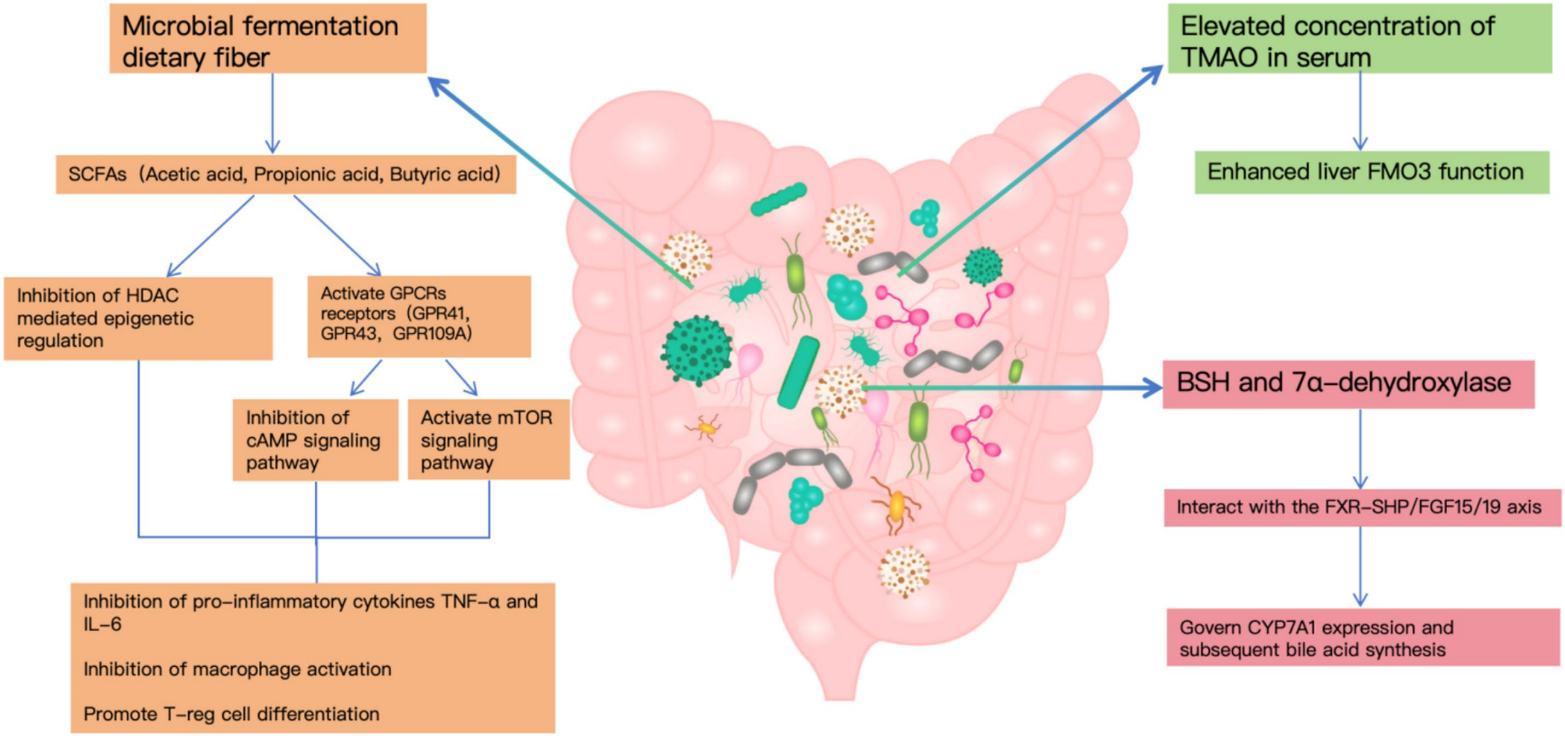
The mechanisms of gut microbiota metabolism and cholesterol stone formation.

Complementary findings from microbial metabolomics have revealed distinct metabolic changes in the feces of these patients. For instance, Chen et al., through liquid chromatography-mass spectrometry (LC–MS), reported a notable reduction in short-chain fatty acids (SCFAs), especially butyrate, accompanied by an increase in secondary bile acids. These shifts were closely linked to changes in the gut microbial composition (15). Furthermore, Wells et al. identified elevated levels of 7α-dehydroxylating bacteria in gallstone patients using fecal dilution techniques. This suggests that cholesterol gallstone formation is strongly associated with a higher proportion of deoxycholic acid in the bile acid pool (16). These findings underscore the intricate relationship between gut microbiota functions and the metabolic pathways underlying gallstone disease (Figure 1).

## 3 Mechanisms of gut microbiota metabolism and cholesterol gallstone formation

The formation of cholesterol gallstones involves sophisticated interactions with gut microbiota metabolism. These microbial communities maintain bile acid homeostasis through an interconnected network encompassing bile acid metabolism, cholesterol processing, and immunological regulation under physiological conditions.

In the context of cholelithiasis development, gut bacterial metabolites—particularly SCFAs, TMAO, and secondary bile acids—emerge as pivotal factors. Microbial fermentation generates SCFAs that influence lipid homeostasis and inflammatory processes through GPCR and HDAC-mediated pathways (15). Notably, individuals with gallstone disease demonstrate markedly increased TMAO concentrations in their serum, corresponding to heightened hepatic FMO3 functionality, which indicates TMAO’s significance in stone development (17).

The gut microbiome’s influence extends to bile acid transformation, primarily through bacterial enzymes BSH and 7α-dehydroxylase, with BSH expression occurring across multiple bacterial genera while 7α-dehydroxylase remains confined to specific anaerobes. These metabolic pathways interact with the FXR-SHP/FGF15/19 axis to govern CYP7A1 expression and subsequent bile acid synthesis (18).

Furthermore, clinical observations reveal substantial shifts in BSH-producing bacterial populations among cholelithiasis patients, contributing to altered bile acid metabolism patterns that significantly impact gallstone formation (8).

Disruption of cholesterol metabolism is a direct cause of gallstone formation. Studies have shown that gut microbiota influence cholesterol absorption and metabolism through multiple pathways. On the one hand, SCFAs produced by gut microbiota suppress hepatic cholesterol synthesis by activating the AMPK signaling pathway (19). On the other hand, certain bacterial species directly participate in cholesterol transformation, impacting its intestinal absorption (20). Additionally, elevated serum levels of trimethylamine N-oxide (TMAO) have been shown to enhance cholesterol secretion into bile, thereby increasing the risk of cholesterol gallstone formation (21).

Inflammatory responses play a crucial role in gallstone formation. Dysbiosis of the gut microbiota disrupts intestinal barrier function, promotes endotoxin translocation, and triggers systemic inflammation. Studies have identified a significant increase in pro-inflammatory bacterial populations and a marked reduction in butyrate-producing anti-inflammatory bacteria in the gut of gallstone patients. This altered microbial composition further exacerbates the pathological process of gallstone formation by influencing immune cell differentiation and function (22).

Microbial metabolites are also pivotal in the pathogenesis of gallstone disease. Beyond SCFAs and bile acids, recent studies have highlighted various microbial-derived metabolites contributing to gallstone formation. For instance, tryptophan metabolites regulate bile acid synthesis via the aryl hydrocarbon receptor (AHR) signaling pathway (23), while sphingolipid metabolites participate in gallstone formation by modulating the expression of cholesterol transport proteins (24). These discoveries provide new perspectives on the mechanisms underlying gallstone pathogenesis.

The relationship between gut microbiota and gallstone formation is marked by intricate bidirectional regulation. While changes in gut microbial composition can drive gallstone development, the presence of gallstones simultaneously impacts the structure and function of the gut microbiota. This dynamic interaction fosters a positive feedback loop that likely contributes to the long-term progression of gallstone disease (25).

## 4 The impact of cholecystectomy on gut microbiota

Cholecystectomy, a widely practiced surgical approach for managing gallstone disease, not only reshapes bile secretion dynamics but also exerts profound effects on the gut microbiome. Continuous bile flow after surgery significantly modifies the concentration and composition of intestinal bile acids, thereby impacting the gut microbial community (26). In the initial postoperative phase (1–3 months), gut microbiota diversity declines substantially. Opportunistic pathogens like Prevotella (27, 28) and *Escherichia coli* (29) become more abundant, while beneficial microbes such as Faecalibacterium (29) and Roseburia (12) diminish. Concurrently, *Clostridium difficile*, a notable opportunistic pathogen, shows a sharp increase. These microbiota shifts correlate strongly with postoperative issues, including diarrhea and indigestion (30).

Surgical intervention triggers metabolic shifts that significantly influence microbial dynamics. Postoperative bile acid changes include elevated levels of free bile acids, especially deoxycholic acid (DCA) and lithocholic acid, accompanied by reduced proportions of conjugated bile acids (31). Such metabolic shifts selectively hinder the proliferation of specific anaerobic bacteria, disturbing the balance of the gut microbiome. Notably, heightened DCA concentrations markedly suppress beneficial butyrate-producing bacteria (32).

Surgery also affects the intestinal immune microenvironment. Single-cell sequencing analyses have revealed significant changes in the immune cell composition of the intestinal mucosa following cholecystectomy. Specifically, the proportion of regulatory T cells (Tregs) decreases, while pro-inflammatory Th17 cells increase. This disruption of immune balance is closely associated with impaired intestinal barrier function and systemic inflammatory responses (33). Studies indicate that these immune alterations partly result from reduced production of tryptophan metabolites by the gut microbiota, which play a crucial role in maintaining intestinal immune homeostasis (34).

In addition, long-term follow-up studies have shed light on the characteristics of gut microbiota recovery after surgery. Zhou et al. conducted a 5-year follow-up study on 20 patients post-cholecystectomy and found that approximately 60% of patients achieved partial gut microbiota recovery within 6 months after surgery. Notably, the study revealed that patients with higher preoperative gut microbiota diversity experienced faster recovery, suggesting that preoperative microbiota status may be an important predictor of postoperative recovery (35).

## 5 Gut microbiota-based strategies for the prevention and treatment of gallstone disease

Recently, therapeutic strategies aimed at modulating the gut microbiota have shown great promise in preventing and managing gallstone disease. These approaches include probiotics, microbial metabolite supplementation, dietary adjustments, and innovative microecological therapies, presenting new opportunities for research and treatment in clinical settings.

Probiotics, as a direct method to regulate gut microbiota, have been thoroughly investigated for their mechanisms and effectiveness. Certain probiotic strains act through diverse mechanisms to inhibit gallstone development. For instance, Lactobacilli and Bifidobacteria with elevated bile salt hydrolase (BSH) activity help maintain bile acid metabolic balance (36). Additionally, butyrate-producing bacteria improve intestinal barrier function and regulate immune responses through SCFAs production (37). These insights underline the potential role of probiotics in gallstone prevention and therapy.

Another promising approach involves targeted supplementation with microbial metabolites. Studies reveal that metabolites like tauroursodeoxycholic acid (TUDCA) and butyrate have notable preventive and therapeutic benefits. TUDCA promotes gallbladder motility and reduces bile stasis by activating G protein-coupled bile acid receptor 1 (GPBAR1), while butyrate enhances intestinal barrier integrity and modulates immunity through GPR43 and GPR109A pathways (38). These findings pave the way for the development of innovative therapeutic drugs.

Dietary interventions have unique advantages in the prevention of gallstone disease. A high-fiber diet significantly promotes the proliferation of probiotics and increases SCFA production. Studies suggest that increasing dietary fiber intake enhances the abundance of butyrate-producing bacteria and reduces the risk of gallstone formation. Additionally, optimizing fatty acid intake by increasing the proportion of unsaturated fatty acids helps maintain a healthy gut microbiota (39). This diet-based intervention strategy is safe, cost-effective, and sustainable.

Microbial replacement therapy has emerged as a promising area of research. Researchers have developed innovative microbiota delivery systems using pH-sensitive coatings to ensure functional microbiota bypass gastric acid and effectively colonize the small intestine. Animal studies have confirmed that this approach significantly enhances cholesterol metabolism and lowers the risk of gallstone formation (40). The advancement of this technology provides new possibilities for precision microecological therapy.

Individualized treatment strategies represent the future trend. By integrating multi-omics data (including metagenomics and metabolomics) with artificial intelligence, Esen et al. developed a personalized gallstone risk prediction model. This precision medicine-based approach enables the design of personalized prevention and treatment strategies tailored to the specific gut microbiota profile of each patient, thereby improving clinical outcomes (41).

Fecal microbiota transplantation (FMT) has also shown potential as an emerging therapeutic approach. Hu et al. observed that gallstone patients treated with FMT experienced significantly enhanced gut microbiota diversity and improved bile acid metabolism (8). However, the long-term efficacy and safety of FMT in gallstone treatment require validation through large-scale, long-term clinical studies.

## 6 Prospects

With advancements in microbiome research and emerging technologies, gallstone prevention and treatment are entering the era of precision medicine. Future research will focus on the following areas:

Multi-omics integration will offer new perspectives for gallstone prevention and treatment. Integrating metagenomics, metatranscriptomics, and metabolomics data with artificial intelligence deep learning algorithms will enable a more comprehensive understanding of the role of gut microbiota in gallstone formation. This systems biology approach is expected to identify novel therapeutic targets and provide scientific evidence for the development of individualized treatment plans (42).

Furthermore, advancements in microbiome engineering have provided new tools for precision interventions. CRISPR-based microbiota editing systems are under development, enabling precise regulation of specific functional genes to alter microbial metabolic functions. Preliminary studies suggest that targeted editing of key metabolic pathway genes can effectively regulate cholesterol metabolism, offering novel intervention strategies for gallstone treatment (43).

In-depth research on the interaction mechanisms between gut microbiota and bile components will uncover new therapeutic targets. Particularly, studies focusing on bile acid signaling pathways and immune regulatory networks are expected to identify additional key regulatory molecules, offering critical insights for the development of innovative therapeutic drugs (44).

International collaborative research is also driving progress in standardizing protocols. Large-scale cohort studies led by international consortia aim to construct microbiome profiles for gallstone disease across diverse populations. These efforts will help establish unified diagnostic and therapeutic standards, providing robust scientific support for precision medicine (45, 46).

Integrative approaches combining traditional Chinese and Western medicine offer new perspectives for optimizing treatment strategies. Modern technologies that clarify the mechanisms of traditional Chinese medicine, particularly its regulation of gut microbiota, may yield more effective combined therapies. This integrative approach has already shown unique advantages in clinical practice (47).

Despite these advancements, challenges remain in gallstone microecological research. Issues such as achieving stable colonization of functional microbiota, optimizing individualized treatment plans, and evaluating long-term efficacy require further investigation. Multidisciplinary collaboration will be essential to achieving more precise and effective prevention and treatment of gallstone disease.

## 7 Conclusion

Gallstone disease is a multifactorial condition influenced by genetic, metabolic, lifestyle, and dietary factors, with gut microbiota playing a pivotal role in its pathogenesis. Recent advancements in high-throughput sequencing and multi-omics technologies have provided profound insights into the interaction between gut microbiota and gallstone formation. Dysbiosis in gallstone patients alters bile acid metabolism, immune regulation, and cholesterol processing, thereby exacerbating disease progression. This understanding has paved the way for innovative prevention and treatment strategies.

Microbiota-targeted approaches, such as probiotics, microbial metabolite supplementation, dietary interventions, and FMT, hold significant promise in reducing gallstone formation risk and improving therapeutic outcomes. Probiotics and microbial metabolites like TUDCA and butyrate have shown notable benefits in regulating bile acid metabolism and enhancing gut barrier integrity. Dietary adjustments, including high-fiber and unsaturated fatty acid intake, provide safe, cost-effective, and sustainable prevention options. Additionally, traditional Chinese medicine, when integrated with Western approaches, offers synergistic effects in gut microbiota modulation.

Emerging technologies, such as CRISPR-based microbiota editing and pH-sensitive microbial delivery systems, represent exciting avenues for precision interventions. These tools enable targeted manipulation of microbial metabolic pathways and effective colonization of therapeutic microbiota, addressing some of the critical challenges in gallstone treatment. Furthermore, personalized medicine approaches leveraging multi-omics data and artificial intelligence are poised to transform clinical management, enabling individualized prevention and therapy.

Despite these advances, challenges persist in achieving stable microbiota colonization, optimizing treatment strategies, and evaluating long-term efficacy. The integration of multi-omics technologies, international collaborations, and multidisciplinary research will be critical to addressing these gaps. Continued exploration of gut microbiota’s interaction with bile acids and immune networks will likely reveal novel therapeutic targets and refine our understanding of gallstone pathogenesis. With these efforts, the prevention and treatment of gallstone disease are poised to enter a new era of precision medicine, offering improved outcomes for diverse patient populations.
